# Birth defects in Brazil: Outcomes of a population-based study

**DOI:** 10.1590/1678-4685-GMB-2018-0186

**Published:** 2020-02-10

**Authors:** Camila Ive Ferreira Oliveira-Brancati, Valéria Cristina Carvalho Ferrarese, Antonio Richieri Costa, Agnes Cristina Fett-Conte

**Affiliations:** 1 Faculdade de Medicina de São José do Rio Preto (FAMERP/FUNFARME), São José do Rio Preto, SP, Brazil.; 2 Universidade de São Paulo (USP), Hospital de Reabilitação de Anomalias Craniofaciais, Bauru, SP, Brazil.

**Keywords:** Malformation, congenital anomaly, fetal development, genetic counseling, public health

## Abstract

Birth defects (BDs) are functional and structural alterations in embryonic or fetal development. With an incidence of approximately 3-5%, BDs are a leading cause of infant mortality and lifelong disability. A population-based prospective case-control study was conducted for one year with 5204 infants, between March 1^st^, 2011 and February 29^th^, 2012 in the city of São José do Rio Preto, State of São Paulo, Brazil. The incidence of BDs was 3.2% [95% confidence interval (95%CI): 2.8-3.8%]. The most common congenital anomalies were heart diseases in isolation (11.2%; 95%CI: 7.3-16.9%) followed by Down syndrome (9.5%; 95%CI: 5.9-14.8%), neural tube defects (8.9%; 95%CI: 5.4-14.1), urinary tract anomalies (7.7%; 95%CI: 4.4-12.7%), and polydactyly (7.0%; 95%CI: 4.0-12.0%). The majority of mothers with Down syndrome babies had advanced age. Family members with the same BD, maternal alcohol consumption, gestational diabetes, and previous miscarriages were the most frequent risk factors. The results were similar to published data from other countries except for the incidence of Down syndrome, which was twice as high as reported by other authors and is probably due to the high sociocultural level of the region where the current study was performed, leading to pregnancies at older maternal age.

## Introduction

Birth defects (BDs) are functional and structural alterations in embryonic or fetal development resulting from genetic, environmental, or unknown causes. BDs have a significant impact on the health and development of a child. They contribute to 50% of neonatal deaths, besides being a leading cause of infant mortality and lifelong disabilities (Gill *et al.*, 2012; Arioglu Aydin *et al.*, 2015). About eight million babies worldwide are born each year with significant BDs (Reece, 2012); the overall incidence is approximately 3-5% (Oliveira *et al.*, 2011; Gill *et al.*, 2012). Although there are well-established risk factors, the causes of most BDs remain unknown (Miller *et al.*, 2011; Kong *et al.*, 2012; Wiener-Megnazi *et al.*, 2012; Ooki, 2013).

While there are many published studies about BDs, few have been performed in underdeveloped and developing countries, with the majority being hospital-based and not population-based studies (Groisman *et al.*, 2013). This article describes data on the incidence and risk factors of BDs obtained in a population-based study performed over one year involving 5204 Brazilian infants.

## Subjects and Methods

This study was approved by the Research Ethics Committee of the Medical School of Sao Jose do Rio Preto (FAMERP # 5838). The parents of all subjects were informed about the nature of the study and signed informed consent forms.

This population-based, prospective, case-control study collected data on all infants born in the city of São José do Rio Preto over one year, between March 1, 2011 and February 29, 2012. Located in the northwestern region of the state of São Paulo, Brazil, the city has a population of 408,258 and, according to the Instituto Brasileiro de Geografia e Estatística, it is the second most developed city in Brazil (IBGE, 2016).

The investigation was performed by a multidisciplinary team involving several kinds of health professionals, in particular neonatologists and geneticists, in all six hospitals of the city. In order to verify the populational character of the study, a partnership with the Health Department of São José do Rio Preto was made. Thus, births of children with BDs carried out in municipalities other than that of the mothers residing in the city could also be considered resident and characterized. However, during the study period, there was no birth of BD babies in a locality other than São José do Rio Preto, according to the official records. In addition, babies born in the city, whose families resided in other municipalities were excluded.

During the study period, 5204 children were born and all were investigated for the presence of major and minor defects. The data of children with BDs (BD Group) were compared with data of healthy infants matched for gender and born in the same hospital immediately after each study case (Control Group). This Group was used to compare maternal and paternal data and obstetric and perinatal variables. Deaths were considered stillbirths when they occurred after 20 weeks of gestation (O’Neill *et al.*, 2011). All cases of BDs were analyzed following the classification of Garne *et al.* (2011).

Trained researchers collected the data by face-to-face interviews with the parents and from medical records. Variables included sociodemographic data, obstetric history, family antecedents, previous pregnancies, periconceptional folate supplementation, gestational exposure to teratogenic agents, prenatal data, type of delivery, birth weight, birth stature, gestational age and results of exams (neonatal screening for metabolic diseases, radiographic evaluations, karyotype, etc.). Samples of peripheral or umbilical cord blood were collected for a karyotype study when indicated in order to confirm or investigate chromosomal abnormalities. Other genetic tests were performed depending on the needs of each individual.

Results were stratified according to BDs and epidemiological parameters. Statistical analysis was performed using the Minitab (version 16) and GraphPad statistical programs. Descriptive statistics using frequencies and proportions were employed to describe the demographic characteristics of subjects. The effects were evaluated by 95% confidence intervals (95%CIs). Statistical analyses included the paired t-test, and Mann-Whitney, chi-square, and Fischer’s exact tests, adopting a level of significance of 5%.

## Results

One hundred and sixty-nine individuals were identified as having BDs revealing an incidence of 3.2% (95% CI: 2.8-3.8%). Table 1 shows the characteristics of subjects with birth defects.

**Table 1:** Characteristics of subjects with birth defects.

Factors	Cases (%)	95% CI
Live Births	158 (93.5%)	88.7-96.3%
Stillbirths	11 (6.5%)	3.7-11.3%
Male	95 (56.2%)	48.7-63.5%
Female	66 (39.0%)	32.0-46.6%
Undetermined	8 (4.7%)	2.4-9.0%

CI = exact confidence interval.

The gestation age of babies with BDs ranged from 20 to 42 weeks [mean: 36.0 weeks; standard deviation (SD): 3 weeks and 6 days]. With respect to the gestational age, 41.2% (95%CI: 34.0-48.8%) were preterm (<37 weeks) and 58.2% (95%CI: 37.2-60.0%) were born at term (≥37 weeks). The majority of children (92.3%; 95%CI: 85.9-97.2%) were delivered by C-section and 42.0% (95%CI: 28.4-49.6%) had low birth weights (<2500 g).

For the Control Group, the period of gestation ranged from 33 to 41 weeks (mean: 38 weeks; SD: 1 week and 2 days), 16.6% (95%CI: 13.4-31.5%) were preterm and 83.4% (95%CI: 68.5-86.6%) were born at term. The majority (93.0%; 95%CI: 87.4-98.0%) were delivered by C-section and 14.7% (95%CI: 10.2-20.9%) had low birth weights (<2500 g). Differences between the groups were statistically significant in respect to the period of gestation (*p*-value = 0.0) and birth weight (p-value = 0.0). There was no significant difference observed in the frequencies of C-sections (*p*-value = 0.8).

Karyotype alterations were detected in 25 individuals; 0.48% (95%CI: 0.33-0.71%) of the total sample (5204 individuals) and 14.8% (95%CI: 10.2-20.9%) of the individuals with BDs (169 individuals). The most common finding was chromosome 21 trisomy (16 cases - 64.0%; 95%CI: 44.5-79.7%), followed by chromosome 13 trisomy (5 cases - 20.0%; 95%CI: 8.9-39.1%), chromosome 18 trisomy (2 cases - 8.0%; 95%CI: 2.2-25.0%), X chromosome monosomy (1 case - 4.0%; 95%CI: 0.7-19.5%) and del(8)(pter→q24:) (1 case - 4.0%; 95%CI: 0.7-19.5%).

The frequencies of BDs are listed in Table 2. Isolated heart diseases (11.2%; 95%CI: 7.3-16.9%) were the most common BDs followed by Down syndrome (9.5%; 95%CI: 5.9-14.8%), neural tube defects (8.9%; 95%CI: 5.4-14.1%), urinary tract anomalies (7.7%; 95%CI: 4.4-12.7%), and polydactyly (7.0%; 95%CI: 4.0-12.0%). According to the classification of Garne *et al.* (2011), isolated anomalies were more frequent, followed by chromosomal syndromes, monogenic diseases, multiple congenital anomalies, and environmental diseases.

**Table 2:** Test for independent interaction of SNPs with LOAD.

Polymorphism	AD Patients N (%)	Controls N (%)	OR (95% IC)	*p*-value^a^	OR (95% IC)	*p*-value^b^
*ABCA7* (rs3764650)						
T	52 (68.4%)	105 (75.5%)	1 (Reference)	-	1 (Reference)	-
G	24 (31.6%)	34 (24.5%)	1.425 (0.767-2.648)	0.262	1.552 (0.794-3.037)	0.199
*CLU* (rs11136000)						
C	89 (57.1%)	170 (58.6%)	1 (Reference)	-	1 (Reference)	-
T	67 (42.9%)	120 (41.4%)	0.938 (0.632-1.390)	0.764	0.840 (0.537-1.314)	0.445
*BIN1* (rs744373)						
T	106 (67.1%)	186 (65%)	1 (Reference)	-	1 (Reference)	-
C	52 (32.9%)	100 (35%)	0.912 (0.605-1.377)	0.662	0.960 (0.606-1.520)	0.861
*APOE* (rs429358 rs7412)						
ε4 -	103 (65.2%)	245 (84.5%)	1 (Reference)	-	1 (Reference)	-
ε4 +	55 (34.8%)	45 (15.5%)	2.907 (1.842-4.588)	<0.001	3.029 (1.873-4.898)	<0.001 ^c^

ε4 - = ε4 non-carriers; ε4+ = ε4 carriers; AD Patients = Alzheimer’s disease patients; OR = odds ratio; CI = confidential interval;*p*-value considered ≤ 0.05; ^a^crude *p*-value; ^b^*p*-value adjusted by the variables age, gender, educational attainment, ethnic background and APOE ε4 status; ^c^ = *p*-value adjusted by the variables on ^b^ except for APOE ε4 status.

Table 3 lists the frequencies of risk factors that were observed in controls and affected children. The frequencies of family members with the same BD (cleft lip/palate - 4 cases, polydactyly - 2 cases, and abnormality of the 3rd toe bilaterally - 1 case), cases of maternal alcohol consumption, gestational diabetes, and previous miscarriages were statistically different between the two groups. The intake of periconceptional folate by mothers of children with BDs and controls was 21.6% and 28.4%, respectively, which was not statistically different between the two groups.

**Table 3:** Characteristics of subjects and their relationship with birth defects.

Factors	Cases N (%)	Controls N (%)	p	OR	95%CI
Parental consanguinity	N=167	N=169			
No	164 (98.2)	169 (100)	0.210	-	0-2.38
Yes	3 (1.8)	0 (0)			
Family history of the same birth defect	N=167	N=169			
No	160 (95.8)	169 (100)	0.007*	-	0-0.67
Yes	7 (4.2)	0 (0)			
Maternal alcohol consumption	N=165	N=169			
No	144 (87.3)	162 (95.9)	0.008*	3.37	1.39-8.17
Yes	21 (12.7)	7 (4.1)			
Gestational diabetes	N=167	N=169			
No	158 (94.6)	168 (99.4)	0.023*	9.56	1.19-76.4
Yes	9 (5.4)	1 (0.6)			
Previous miscarriage	N=167; %	N=169; %			
No	132 (79.0)	155 (91.8)	0.000*	2.93	1.51-5.69
Yes	35 (21.0)	14 (8.2)			
Twin pregnancy	N=169	N=169			
No	158 (93.5)	166 (98.2)	0.056	3.85	1.05-14.0
Yes	11 (6.5)	3 (1.8)			

OR=odds ratio; CI = exact confidence interval; *statistically significant.

The mean maternal age for mothers of babies with BDs was 28.0 years (95%CI: 26.9-29.2%; range: 14-45 years; SD: 7.3 years) and for control mothers was 26.0 years (95%CI: 25.0-26.9%; range: 14-41 years; SD: 6.2 years). Of the mothers of babies with BDs, 66.0% (95%CI: 58.6-72.8%) were aged from 20 to 34 years, 19.6% (95%CI: 14.3-26.3%) were 35 years old or more, and 14.3% (95%CI: 9.8-20.4%) were under 19 years old. Among the Control Group mothers, 74.0% (95%CI: 66.8-80.0%) were aged from 20 to 34 years, 17.7% (95%CI: 12.7-24.2%) were under 19 years old, and 8.3% (95% CI: 5.0-13.4%) were 35 years old or more. Significantly more mothers of affected children had advanced age (≥ 35) than in Control Group mothers (*p*-value = 0.008). Forty-six percent (95%CI: 27.9-64.9%) of the mothers of children with numerical chromosomal abnormalities were at least 35 years old.

The mean age of the fathers of babies with BDs was 31.5 years old (95%CI: 30.2-32.7%; range: 17-54 years; SD: 7.9 years) and for the Control Group fathers it was 29.6 years (95%CI: 28.6-30.6%; range: 17-49 years; SD: 6.5 years) Advanced age (≥ 40) was seen in 15% (95%CI: 9.8-21.0%) of the fathers of the BD Group and 6% (95%CI: 2.8-10.6%) of the Control Group (p-value = 0.016). None of the fathers of children with monogenic syndromes was over 40 years old.

## Discussion

Studies of BDs should address the collection, analysis, and dissemination of information and contribute to local interventions such as prevention, diagnosis, and treatment. However, in many low- and middle-income countries, BDs are not considered a public health priority and are perceived by the medical community as rare, unpreventable events. There are few studies performed in these countries and most are usually hospital-based (Groisman *et al.*, 2013). In Brazil, the situation is not different, and this population-based study can be considered an example of the BDs reality in a country that has a highly mixed population with broad cultural diversity (IBGE, 2016).

Although the incidence of BDs around the world is variable, most studies report 3-5% in different populations (Oliveira *et al.*, 2011). The incidence in this study (3.2%) corroborates previously described data despite the differences in diagnostic levels, sample sizes, and methodologies of other studies performed both in Brazil (1.4-5%) and other countries (Oliveira *et al.*, 2011; Gill *et al.*, 2012; Naim *et al.*, 2013). The incidence of BDs was slightly higher in males than in females as has previously been reported(Asindi *et al.*, 1997).

Many infants with BDs have low birth weights and are premature. Globally, an estimated 13 million babies are born annually before completing 37 weeks of gestation with the rates generally being the highest in low- and middle-income countries. Even so, preterm birth rates vary greatly within countries and are related to sociodemographic characteristics (Lawn *et al.*, 2010). In the current study population, the frequency of premature births and babies weighing less than 2500g was high compared to the world literature (Lawn *et al.*, 2010; Herbert *et al.*, 2012). Ordinarily, much higher incidences of congenital anomalies are observed among children who weigh 2500g or less at birth than among those who are heavier (Herbert *et al.*, 2012).

Generally, the rate of C-sections is related to prematurity and low weight as they are performed for convenience reasons. Elective preterm C-sections are still largely practiced in cases of prenatal diagnoses of major congenital anomalies (Calisti *et al.*, 2012). The C-section was the commonest mode of delivery in both the BD and Control Groups. This was expected because it is common for parents to opt for this kind of delivery in Brazil, regardless of the fetal status (Freitas *et al.*, 2015).

Most chromosomal defects are lethal and identified after miscarriages. They account for a high rate of congenital anomalies. For example, trisomies of chromosomes 13, 18, and 21 and X chromosome monosomy have an impact on births (~0.3% of all births; Wiener-Megnazi *et al.*, 2012), and their frequency was similar in the current study. However, the trisomy of chromosome 21 (the second most common BD observed here) affects approximately 1 in 660 births (Hwang and Jea, 2013) with the incidence in the current study being 2 in 660, twice as high as generally described. This could be explained by the significant advanced age observed among mothers; it should also be considered that abortion is illegal in Brazil.

Congenital heart diseases are the most common BDs in humans. They can be isolated or one of multiple defects (Weber, 2012; Zaidi *et al.*, 2013). In this study, heart diseases in isolation were the most common BD. The frequency of neural tube defects ranges from 0.2 to 10 per 1000 live births in specific geographical locations (Copp *et al.*, 2013). In this study, this was the third most common BD. Other BDs, such as congenital anomalies of the kidneys and urinary tract, and polydactyly, the fourth and fifth most common findings, respectively, are among the most common anomalies in newborn infants (Richter-Rodier *et al*.; 2012; Liu *et al.*, 2016).

The incidence of BDs not only reflects allelic frequencies of deleterious genes in a population but it is also affected by environmental factors (Alaani *et al.*, 2011; Naim *et al.*, 2012). Of the known risk factors for congenital abnormalities that were investigated in this study, the frequencies of familial recurrence, maternal alcohol consumption, gestational diabetes, previous miscarriages and advanced maternal age were significantly higher in the BD Group than the Control Group.

Similar to previous descriptions, certain BDs in this study recurred in families, such as non-syndromic oral cleft, polydactyly and abnormalities in the position of fingers (Jones, 2007; Grosen *et al.*, 2010; Ravichandran *et al.*, 2012).

Three times more mothers of the BD Group consumed alcohol during pregnancy. In Brazil, as in other countries, this is a health problem (Feldman *et al*., 2012; Veloso and Souza Monteiro, 2013). In the United States, approximately 80,000 women annually consume alcoholic beverages through all three trimesters of pregnancy. Prenatal alcohol exposure is a risk factor for BDs and can lead to a wide range of adverse effects in a developing fetus. Fetal alcohol spectrum disorder is a developmental disorder that affects up to 0.2% of births (Bates, 2013). The risk of comorbid disorders is also increased for this population; this increases the phenotype severity and complexity of management (Paintner *et al.*, 2012; Viteri *et al.*, 2015).

The rate of gestational diabetes mellitus was significantly different between the groups. This is believed to be a risk factor as it has been correlated to limb and heart defects, as observed in this study. Epidemiologic studies have shown that low glycemic dietary management can reduce the incidence of BDs (Reece, 2012; Glover *et al.*, 2016).

Recurrent pregnancy loss affects 2-5% of all couples with >50% being characterized as unexplained or idiopathic (Hodes-Wertz *et al.*, 2012). The results of this study showed that the rate of previous miscarriages in mothers of the BD Group was significantly higher. Indeed, previous miscarriages are considered to be a risk factor for future babies to have some type of BD (Lu *et al.*, 2011; Machado *et al.*, 2013).

More women are delaying child bearing, which results in an annual rate of 14.9% of live births to women aged 35 years or older (Hamilton *et al*., 2010). Both younger and older maternal ages may pose increased risks for BDs (Miller *et al.*, 2011; Gill *et al.*, 2012). In this study, about 20% of mothers of babies with BDs were at least 35 years old. Thus, the effect of advanced maternal age was, as expected, greatly related to BDs, such as Down syndrome. Although advanced paternal age has been associated with an increased risk for congenital disorders (Kong *et al.*, 2012; Wiener-Megnazi *et al.*, 2012; Goriely and Wilkie, 2012), this was not observed in the current study.

There was no significant difference between the BD and Control Groups in relation to the rate of consanguineous marriages and twin pregnancies, considered major risk factors for congenital anomalies (Weller *et al.*, 2012; Boyle *et al.*, 2013; Sheridan *et al.*, 2013; Maghsoudlou *et al.*, 2015); however, one case of Smith-Lemli-Opitz syndrome, an autosomal recessive disorder (Quélin *et al.*, 2012), probably resulted from parental consanguinity.

Neural tube defects were one of the most common BDs observed in this study, as is reported in publications around the world, with a prevalence from 1-10% (Wang *et al.*, 2013). A prudent periconception supplementation with folic acid may decrease the risk, as well as the threat of heart defects and other BDs (Czeizel, 2012; Sotres-Alvarez *et al.*, 2013). In this study, the periconception supplementation did not differ between the groups and was low, below the expected, for a developed region.

Infant mortality is one of the most important conditions to be considered in relation to BDs because it is indicative of the health of a community or country. Early recognition of anomalies is important for planning and care (Calisti *et al.* (2012). For example, congenital disorders continue to be a leading cause of infant mortality and rank within the top 15 contributors to the global burden of disease, even though many congenital disorders are preventable (Shannon *et al.*, 2013). Thus, the identification of incidence, risk factors, and consequences of BDs are essential to plan preventive measures and effective treatment.

Findings involving BDs around the world vary due to the methods of investigation and differ because of the cultural, social, and geographic context of each population. No similar study for Brazil was found, i.e., involving birth data of all infants born in a municipality over a period of time. The studies on BDs are usually performed with a hospital-based sample of births. Even the Latin-American collaborative study of congenital malformations (ECLAMC), a program for the clinical and epidemiological investigation of risk factors in the etiology of congenital anomalies in Latin-American hospitals (Castilla and Orioli, 2004), offers limited information, since the number of participant hospitals is still small (Guerra *et al.*, 2008).

Based on these preliminary findings, this population-based study performed in Brazil can collaborate in developing strategies for the healthcare and education needs of the population. The incidence of BDs reported was similar to published data from other countries, but the incidence of Down syndrome was twice as high as the rate reported in the literature. This can be the result of the reality in the region where the study was performed. The region is economically more advanced and many women delay child bearing, increasing the risk for congenital abnormalities. Family history of the same BDs, maternal alcohol consumption, gestational diabetes, and previous miscarriages were the more frequent risk factors. In this context, different strategies should be developed to reduce the occurrence of BDs depending on the current situation of congenital defects in different regions.

## References

[B1] Alaani S, Savabieasfahani M, Tafash M, Manduca P (2011). Four polygamous families with congenital birth defects from
Fallujah, Iraq. Int J Environ Res Public Health.

[B2] Arioglu Aydin Ç, Aydin S, Serdaroglu H (2016). Multifetal gestations with assisted reproductive technique before
the single-embryo transfer legislation: obstetric, neonatal outcomes and
congenital anomalies. J Matern Fetal Neonatal Med.

[B3] Asindi AA, Al Hifzi I, Bassuni WA (1997). Major congenital malformations among Saudi infants admitted to
Asir Central Hospital. Ann Saudi Med.

[B4] Bates EA (2013). A potential molecular target for morphological defects of fetal
alcohol syndrome: Kir2.1. Curr Opin Genet Dev.

[B5] Boyle B, McConkey R, Garne E, Loane M, Addor MC, Bakker MK, Boyd PA, Gatt M, Greenlees R, Haeusler M (2013). Trends in the prevalence, risk and pregnancy outcome of multiple
births with congenital anomaly: A registry-based study in 14 European
countries 1984-2007. BJOG.

[B6] Calisti A, Oriolo L, Giannino G, Spagnol L, Molle P, Buffone EL, Donadio C (2012). Delivery in a tertiary Center with co-located surgical facilities
makes the difference among neonates with prenatally diagnosed major
abnormalities. J Matern Fetal Neonatal Med.

[B7] Castilla EE, Orioli IM (2004). ECLAMC: the Latin-American collaborative study of congenital
malformations. Community Genet.

[B8] Copp AJ, Stanier P, Greene ND (2013). Neural tube defects: recent advances, unsolved questions, and
controversies. Lancet Neurol.

[B9] Czeizel AE (2012). Experience of the Hungarian Preconception Service between 1984
and 2010. Eur J Obstet Gynecol Reprod Biol.

[B10] Feldman HS, Jones KL, Lindsay S, Slymen D, Klonoff-Cohen H, Kao K, Rao S, Chambers C (2012). Prenatal alcohol exposure patterns and alcohol-related birth
defects and growth deficiencies: a prospective study. Alcohol Clin Exp Res.

[B11] Freitas PF, Drachler ML, Leite JC, Grassi PR (2005). Social inequalities in cesarean section rates in primiparae,
Southern Brazil. Rev Saude Publ.

[B12] Garne E, Dolk H, Loane M, Wellesley D, Barisic I, Calzolari E, Densem J, EUROCAT Working Group (2011). Surveillance of multiple congenital anomalies: implementation of
a computer algorithm in European registers for classification of
cases. Birth Defects Res A Clin Mol Teratol.

[B13] Gill SK, Broussard C, Devine O, Green RF, Rasmussen SA, Reefhuis J, National Birth Defects Prevention Study (2012). Association between maternal age and birth defects of unknown
etiology - United States, 1997-2007. Birth Defects Res A Clin Mol Teratol.

[B14] Glover AV, Tita A, Biggio JR, Harper LM (2016). Examining the starting dose of glyburide in gestational
diabetes. Am J Perinatol.

[B15] Goriely A, Wilkie AO (2012). Paternal age effect mutations and selfish spermatogonial
selection: Causes and consequences for human disease. Am J Hum Genet.

[B16] Groisman B, Bidondo MP, Gili JA, Barbero P, Liascovich R (2013). Strategies to achieve sustainability and quality in birth defects
registries: the experience of the national registry of congenital anomalies
of Argentina. J Registry Manag.

[B17] Grosen D, Chevrier C, Skytthe A, Bille C, Mørtensen K, Sivertsen A, Murray JC, Christensen K (2010). A cohort study of recurrence patterns among more than 54,000
relatives of oral cleft cases in Denmark: support for the multifactorial
threshold model of inheritance. J Med Genet.

[B18] Guerra FA, Llerena JC, Gama SG, Cunha CB, Theme Filha MM (2008). Reliability of birth defect data on birth certificates of Rio de
Janeiro, Brazil, 2004. Cad Saude Publ.

[B19] Hamilton BE, Martin JA, Ventura SJ, Division of Vital Statistics (2010). Births: Preliminary Data for 2009. Natl Vital Statist Rep.

[B20] Herbert DL, Lucke JC, Dobson AJ (2012). Birth outcomes after spontaneous or assisted conception among
infertile Australian women aged 28 to 36 years: A prospective,
population-based study. Fertil Steril.

[B21] Hodes-Wertz B, Grifo J, Ghadir S, Kaplan B, Laskin CA, Glassner M, Munné S (2012). Idiopathic recurrent miscarriage is caused mostly by aneuploid
embryos. Fertil Steril.

[B22] Hwang SW, Jea A (2013). A review of the neurological and neurosurgical implications of
Down syndrome in children. Clin Pediatr (Phila).

[B23] Jones KLJ (2007). Padrões reconhecíveis de malformações congênitas. 6th edition.

[B24] Kong A, Frigge ML, Masson G, Besenbacher S, Sulem P, Magnusson G, Gudjonsson SA, Sigurdsson A, Jonasdottir A, Wong WS (2012). Rate of *de novo* mutations and the importance of
father’s age to disease risk. Nature.

[B25] Lawn JE, Gravett MG, Nunes TM, Rubens CE, Stanton C, GAPPS Review Group (2010). Global report on preterm birth and stillbirth (1 of 7):
Definitions, description of the burden and opportunities to improve
data. BMC Pregnancy Childbirth.

[B26] Liu QG, Sun J, Xiao XW, Song GR (2016). Birth defects data from surveillance hospitals in Dalian City,
China, 2006-2010. J Matern Fetal Neonatal Med.

[B27] Lu QB, Wang ZP, Gao LJ, Gong R, Sun XH, Zhao ZT (2011). Previous abortion and the risk of neural tube defects: a
case-control study. J Reprod Med.

[B28] Machado CJ, Lobato AC, Melo VH, Guimarães MD (2013). Spontaneous and voluntary fetal losses in Brazil in 1999-2000: A
study of associated factors. Rev Bras Epidemiol.

[B29] Maghsoudlou S, Cnattingius S, Aarabi M, Montgomery SM, Semnani S, Stephansson O, Wikström AK, Bahmanyar S (2015). Consanguineous marriage, prepregnancy maternal characteristics
and stillbirth risk: A population-based case-control study. Acta Obstet Gynecol Scand.

[B30] Miller A, Riehle-Colarusso T, Siffel C, Frías JL, Correa A (2011). Maternal age and prevalence of isolated congenital heart defects
in an urban area of the United States. Am J Med Genet A.

[B31] Naim A, Al Dalie H, El Balawi M, Salem E, Meziny K, Sharwwa R, Minutolo R, Manduca P (2012). Birth defects in Gaza: Prevalence, types, familiarity and
correlation with environmental factors. Int J Environ Res Public Health.

[B32] Oliveira CIF, Richieri-Costa A, Carvalho Ferrarese VC, Móz Vaz DC, Fett-Conte AC (2011). Birth defects in newborns and stillborns: an example of the
Brazilian reality. BMC Res Notes.

[B33] O’Neill SM, Kearney PM, Kenny LC, Khasan AS, Henriksen TB, Lutomski JE, Greene RA (2013). Caesarean delivery and subsequent stillbirth or miscarriage:
systematic review and meta-analysis. PLoS One.

[B34] Ooki S (2013). Maternal age and birth defects after the use of assisted
reproductive technology in Japan, 2004-2010. Int J Womens Health.

[B35] Paintner A, Williams AD, Burd L (2012). Fetal alcohol spectrum disorders—implications for child
neurology, part 2: diagnosis and management. J Child Neurol.

[B36] Quélin C, Loget P, Verloes A, Bazin A, Bessiéres B, Laquerrière A, Patriere R, Grigorescu R, Encha Razavi F, Delahaye S (2012). Phenotypic spectrum of fetal Smith-Lemli-Opitz
syndrome. Eur J Med Genet.

[B37] Ravichandran K, Shoukri M, Aljohar A, Shazia NS, Al-Twaijri Y, Al Jarba I (2012). Consanguinity and occurrence of cleft lip/palate: A
hospital-based registry study in Riyadh. Am J Med Genet A.

[B38] Reece EA (2012). Diabetes-induced birth defects: What do we know?. What can we do? Curr Diab Rep.

[B39] Richter-Rodier M, Lange AE, Hinken B, Hofmann M, Stenger RD, Hoffmann W, Fusch C, Haas JP (2012). Ultrasound screening strategies for the diagnosis of congenital
anomalies of the kidney and urinary tract. Ultraschall Med.

[B40] Shannon GD, Alberg C, Nacul L, Pashayan N (2013). Preconception health care and congenital disorders: mathematical
modelling of the impact of a preconception care programme on congenital
disorders. BJOG.

[B41] Sheridan E, Wright J, Small N, Corry PC, Oddie S, Whibley C, Petherick ES, Malik T, Pawson N, McKinney PA (2013). Risk factors for congenital anomaly in a multiethnic birth
cohort: an analysis of the Born in Bradford study. Lancet.

[B42] Sotres-Alvarez D, Siega-Riz AM, Herring AH, Carmichael SL, Feldkamp ML, Hobbs CA, Olshan AF, National Birth Defects Preventiomn Study (2013). Maternal dietary patterns are associated with risk of neural tube
and congenital heart defects. Am J Epidemiol.

[B43] Veloso LU, de Souza Monteiro CF (2013). Prevalence and factors associated with alcohol use among pregnant
adolescents. Rev Lat Am Enfermagem.

[B44] Viteri OA, Soto EE, Bahado-Singh RO, Christensen CW, Chauhan SP, Sibai BM (2015). Fetal anomalies and long-term effects associated with substance
abuse in pregnancy: a literature review. Am J Perinatol.

[B45] Wang M, Wang ZP, Gao LJ, Gong R, Sun XH, Zhao ZT (2013). Maternal body mass index and the association between folic acid
supplements and neural tube defects. Acta Paediatr.

[B46] Weber S (2012). Novel genetic aspects of congenital anomalies of kidney and
urinary tract. Curr Opin Pediatr.

[B47] Weller M, Tanieri M, Pereira JC, Almeida Edos S, Kok F, Santos S (2012). Consanguineous unions and the burden of disability: A
population-based study in communities of Northeastern Brazil. Am J Hum Bio.

[B48] Wiener-Megnazi Z, Auslender R, Dirnfeld M (2012). Advanced paternal age and reproductive outcome. Asian J Androl.

[B49] Zaidi S, Choi M, Wakimoto H, Ma L, Jiang J, Overton JD, Romano-Adesman A, Bjornson RD, Breitbart RE, Brown KK (2013). *De novo* mutations in histone-modifying genes in congenital
heart disease. Nature.

